# Designing an iPad App to Monitor and Improve Classroom Behavior for Children with ADHD: iSelfControl Feasibility and Pilot Studies

**DOI:** 10.1371/journal.pone.0164229

**Published:** 2016-10-14

**Authors:** Sabrina Schuck, Natasha Emmerson, Hadar Ziv, Penelope Collins, Sara Arastoo, Mark Warschauer, Francis Crinella, Kimberley Lakes

**Affiliations:** 1 Child Development School, Department of Pediatrics, School of Medicine, University of California Irvine, Irvine, California, United States of America; 2 Department of Information & Computer Sciences, University of California Irvine, Irvine, California, United States of America; 3 School of Education, University of California Irvine, Irvine, California, United States of America; Gentofte Hospital, DENMARK

## Abstract

Children with Attention Deficit/Hyperactivity Disorder (ADHD) receive approximately 80% of instruction in the general education classroom, where individualized behavioral management strategies may be difficult for teachers to consistently deliver. Mobile device apps provide promising platforms to manage behavior. This pilot study evaluated the utility of a web-based application (iSelfControl) designed to support classroom behavior management. iSelfControl prompted students every ‘Center’ (30-minutes) to self-evaluate using a universal token-economy classroom management system focused on compliance, productivity, and positive relationships. Simultaneously, the teacher evaluated each student on a separate iPad. Using Multi Level Modeling, we examined 13 days of data gathered from implementation with 5^th^ grade students (N = 12) at a school for children with ADHD and related executive function difficulties. First, an unconditional growth model evaluated the overall amount of change in aggregated scores over time as well as the degree of systematic variation in scores within and across teacher-student dyads. Second, separate intercepts and slopes were estimated for teacher and student to estimate degree of congruency between trajectories. Finally, differences between teacher and student scores were tested at each time-point in separate models to examine unique ‘Center’ effects. 51% of the total variance in scores was attributed to differences between dyads. Trajectories of student and teacher scores remained relatively stable across seven time-points each day and did not statistically differ from each other. On any given day, students tended to evaluate their behaviors more positively (entered higher scores for themselves) compared to corresponding teacher scores. In summary, iSelfControl provides a platform for self and teacher evaluation that is an important adjunct to conventional classroom management strategies. The application captured teacher/student discrepancies and significant variations across the day. Future research with a larger, clinically diagnosed sample in multiple classrooms is needed to assess generalizability to a wider variety of classroom settings.

## Introduction

In the United States, children with Attention Deficit/Hyperactivity Disorder (ADHD) typically receive at least 80% of their academic instruction in the general education classroom, wherein legally-mandated individualized intervention, support, and accommodation as prescribed by the US Individuals with Disabilities in Education Act (IDEA), may be sporadic [1,2]. One barrier to providing such support may be the perception that individualized interventions are impractical and time consuming [1,3], but even teachers who are willing to go the extra step are often precluded from doing so because of limited understanding of ADHD-related behavioral deficits and insufficient training in how to monitor and implement appropriate intervention [3–5]. These instructional barriers might be overcome via a user-friendly tablet-based application designed to implement evidence-based intervention strategies. The objective of this pilot study was to assess the feasibility and impact of utilizing a web-based application for tablets (iSelfControl) designed to improve children’s self-regulation in the classroom setting.

Assistive technologies are now widely used to help students communicate with others, access information on the internet and enhance the curriculum and augment a wide range of cognitive processes. Gilespie, Best, and O’Neal [6] recently published a systematic review of 91 studies on assistive technologies for cognition conducted since 1972, the majority over the last decade. These studies examined the use of technologies to assist students with time management, organization and planning, attention, experience of self, memory, and emotional regulation. The reviewers found considerable promise in assistive technologies, but “to achieve their potential, they need to be available at the point of need”. They concluded that mobile devices and apps are an especially promising platform for assistive technologies due to their convenience and increased accessibility, both inside and outside the classroom.

Among mobile devices, digital tablets, and in particular the iPad, have proven especially promising for children with learning challenges. The light weight, flexible orientation, instant-on capacity and touch-screen interface of iPads provide an intuitive, engaging, and motivating platform for learners with special needs [7]. They are a welcome replacement for computers, which are cumbersome, as well as for specialized devices, which are typically more expensive and stigmatizing [8]. Though systematic research on iPad use by learners with special needs is just beginning, there is already evidence that iPads have been enthusiastically embraced by children, parents and teachers as feasible to use, and case studies suggest substantial positive impact on special education classrooms from the use of iPads and other iOS devices [9–12]. Whereas much of the research has focused on the use of iPads for building students’ communication skills [13] and academic skills [14–16], only a handful have explored their use to support students’ adaptive behaviors [17]. Studies to date tend to be with a small number of participants and predominantly qualitative and descriptive in nature (Kangohara [18], finding 15 studies after a systematic review of the literature involving the use of such devices for individuals with developmental disabilities; see Edyburn [19] for a description of the adoption of iPads and apps in special education and the lack of rigorous research on technology innovations; see Kiger, Herro, & Prunty [20], for an example of a quasi-experimental study finding a moderate impact of iPod use for math fact proficiency; and Spooner, Kemp-Inman, Ahlgrim-Delzell, Wood, & Davis [21], for a quasi-experimental study using iPads for literacy skills for students with severe disabilities. The present study aims to examine the feasibility and impact of a web-based app developed to utilize self-evaluation and behavior management strategies to improve self-regulation in the classroom.

Our overarching hypothesis was that utilizing iSelfControl directly in the classroom in conjunction with a token economy system would lead to measurable improvements in self-awareness and self-regulation. iSelfControl was designed to support three processes central to building self-regulation. Specifically, we sought to understand if children were better able to (a) monitor their behavior by focusing their attention on the present moment (*What am I doing right now*?); (b) evaluate their behavior (*What am I supposed to be doing right now*?), and (c) correct their behavior, if necessary (*What can I do to meet my goals*?). We further anticipated that the data gathered by the application could be used to inform individualized intervention in the classroom.

Prior to implementing the tool and analyzing the program data, staff and volunteers at a unique school-based behavioral health program designed the application in a collaborative process with ideas and feedback from the classroom students. This feedback was given to the development team who developed the application accordingly. Once it was ready for use, the application was presented to the clinical staff and volunteers (teachers and behavior support specialists), who, in turn, explained it to the students. Students and classroom staff were assigned unique and non-identifying individual user names and passwords to gain access to the web-based application.

**Fig 1:**
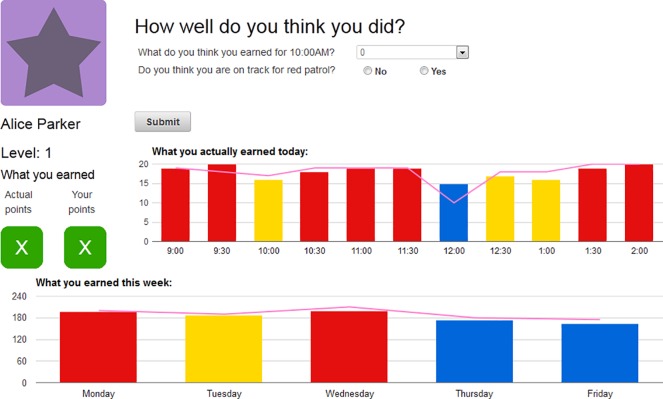
Screen Shot from the Original App (Data and Student Name are Not Real).

Of note, the program in which the app was tested is a facility operated by a public university for children who have challenges in the area of attention, behavior regulation, and interpersonal relationships—primarily children with ADHD. The unique laboratory setting delivers regular education curriculum integrated with a universal token economy behavior management system (based on earned points to reward ‘time on task’ and other adaptive behavior). As part of the standard curriculum and materials implemented at the participating school, each student had an iPad on his/her desk. The iSelfControl application prompted students every 30 minutes through a step-by-step self-evaluation process considering the last 30 minutes of classroom behavior. At the same time, classroom staff also recorded behavioral observations for each student on a separate iPad. After logging their entries, students were able to compare their ratings to those made by the classroom staff, giving them an idea of how consistent their self-evaluation was with that of the classroom staff. Throughout the day, students were able to view their progress on charts displayed by the app directly on the iPad (Fig 1).

### Feasibility Study

Our feasibility study resulted in the development of the first version of iSelfControl with the following features:

#### Targeted goals for adaptive and functional classroom behavior

For every 30 minute period (“center”), students earned points for demonstrating adaptive behaviors key to school success including: ‘Following Directions’, ‘Following Rules’, ‘Staying on Task’, and ‘Getting Along’ with others. Demonstrating maladaptive behaviors reduced the number of points a student earned each center.

#### Unique teacher and student interfaces

As stated earlier, classroom staff and students had unique logins for the web-based application. Every center, the classroom teacher recorded points earned based on observed student behaviors in the domains described above. Students then were tasked with reflecting on their behavior over the center and scored themselves by briefly reporting the number of points they believed they earned in each domain. Students were able to view the teacher’s score for that center only after completing their own entries. The teacher was able to review students’ scores after completing their own entries.

Subsequently, students were asked to identify a step they could have taken to be more present and on task (if necessary). This process is similar to one of the mindfulness exercises used in programs for children, which Hooker & Fodor [22] referred to as “Awareness of Self in the Environment;” the intent of this exercise is to “help the child to pay attention to both the environment and his or her actions, rather than moving through the day like a robot”.

The classroom teacher’s task during this exercise was simply to enter the actual center behavior scores for each child. The app was designed so that the staff interface had drop-down menus allowing them to complete entries for 10 students in about one–two minutes. Of note, on the classroom staff interface, all students are listed on a single screen, to reduce the amount of time needed to make multiple entries.

Moreover, iSelfControl provided students and classroom staff with a mechanism to promote and monitor student self-regulation in the classroom (based on earned points later used to reward adaptive classroom behavior). By aggregating data, the app provided feedback on student progress throughout the day, week, and over longer periods of time. At the end of the day, students could view charts of their progress over the day and were asked to reflect on what they did well and what they would like to improve the next day.

### The Present Study

Following the developmental phase described above, we conducted a pilot study. Our primary goals were to: 1) evaluate the feasibility of both staff and students utilizing a tablet-based application in the classroom setting; 2) determine if students demonstrated improved student self-awareness and self-regulation and 3) determine if information obtained from dyad scores recorded in this application helped to inform classroom behavior management for children with challenges related to ADHD.

## Method

### Clinical/Educational Setting

The study was conducted on archival data collected in the context of the laboratory school setting described above. The iSelfControl application was piloted in one classroom (grade 5) over a six-week period. Upon review by the local Institutional Review Board, because no identifying information was included in the data set analyzed by the investigators, and there existed no link between the archival data to the individuals at the site, this work did not meet criteria as Human Research, but rather was categorized as Programmatic Evaluation, not requiring IRB approval.

### Population

In the course of the school-based behavioral health program, data was collected from all students in Grade 5 (N = 12) and their classroom teacher (N = 1). Students were between the ages of 9–11 years (*M* = 9.75; *SD* = .55), and all students were males. All students who attend this school have histories of significant difficulties in conventional classroom settings that are related to deficits in attention and self-control. As standard procedure in the operations of the facility, all programmatic data is stripped of identifying information (including diagnoses) by the school staff, and then placed into a local data repository where archives can then be accessed and analyzed for the purposes of programmatic evaluation.

### Procedure

Data collection began on the first day of school instruction in the autumn and analyses were conducted with only paired dyadic observations, in which the teacher recorded the student’s points earned for each “center” (30 minute period of the token economy) and the corresponding student also recorded his perception of his points earned. For every 30 minute period (“center”), students earned points for demonstrating adaptive behaviors key to school success including: ‘Following Directions’, ‘Following Rules’, ‘Staying on Task’, and ‘Getting Along’ with others. Demonstrating maladaptive behaviors reduced the number of points a student earned each center. The point system is part of the school program, where the earning of points and subtraction of points for behavior are clearly taught to children.

Data was only included for analysis when each dyad (teacher and student) entered concurrent ratings during the same “center point check” or period of time, resulting in a total of 13 days of dyadic data. The software to run iSelfControl was developed as a Web application, deployed using Google App Engine, and included as its primary components a database implementation, HTML pages, and Python code. This application can run in any Web browser, and can be accessed via each individual classroom iPad privately to a secure and encrypted web-based data source. The code for this software has been deposited in an appropriately accessible archive hosted at UC Irvine and conforms to the Open Source Definition as describe by the PlosOne policies. It is available by contacting lead author.

### Analyses

Multilevel modeling (MLM) analyses were used due to the hierarchical structure of the data in order to adjust for the non-independence (i.e., clustering) of repeated observations nested within the same individual, and thus, allows for the examination of interpersonal and intrapersonal differences in outcomes over time [23]. Extending on individual-level models, in MLM for dyadic data, repeated measures for each dyad member are considered as Level 1 units nested within the Level 2 unit, the dyad. Advantages to using MLM for dyads include estimating unique trajectories for partners; allowing trajectories to be directly tested for differences at the intercept (predicted score at a specified time point) and slope (rate of change); and accounting for the interdependence of partner outcomes within the same dyad [24–25]. Finally, multilevel models do not require balanced data (i.e., the same number or equally spaced waves of time points for each individual or dyad), which was not present in the current sample.

Archival data were analyzed in three phases. First, as a prerequisite for estimating growth models with level-2 predictors, an unconditional growth model was used to evaluate the overall baseline amount of change in aggregated center scores over time as well as the degree of systematic variation in scores within dyads and across dyads. The centers were aggregated over time in efforts to detect improvement (i.e., increased congruity) in the overall trajectories of student and teacher data across a two-week period of time. Points were first aggregated (i.e., averaged) across all centers at the daily level for separate indices of student and teacher daily mean points, which were then aggregated across all 13 days. Aggregating data across many observations at the phase (time) level, person (student vs. teacher) level, and dyadic level allows for greater flexibility and interpretation of data to examine within- and between-dyad variability trends over time. Differences between student and teacher daily mean points at each center, averaged across all 13 days, can also be tested while controlling for the effects from the previous center. Specifically, a time variable was created and centered at the first wave of data collection of each day (center 1) to represent initial baseline status, or when time = 0, and linear time was entered as the sole predictor (no quadratic effect of time was found). When separate trajectories were estimated across dyads (intercepts and slopes treated as random effects), the slope parameter showed near zero variance and problems with model convergence occurred. This, along with significant intercept variance, suggested that the degree of change in scores did not vary across dyads; thus, the linear effect of time was treated as a fixed effect in the final trimmed growth models.

In the second set of analyses, separate intercepts and slopes were estimated for teacher and student partners, along with different variance components, in order to estimate the degree of congruency between student and teacher trajectories (i.e., the covariance between intercepts and slopes). Because two-intercept models do not allow for direct tests of mean-level differences in intercepts and slopes, these models were then re-parameterized such that intercept and slope coefficients were pooled across dyad members and differences across dyad members were tested. Finally, differences between teacher and student scores were tested at each time point in separate models to examine the unique effects of each center on average daily scores.

Additionally, qualitative and quantitative data was collected anonymously from the teacher and students who used the application. This information was collected utilizing a structured consumer satisfaction survey developed specifically for the evaluation of this app in the program.

## Results & Discussion

### Feasibility & Acceptability

The project was piloted over a 6 week period (28 school days), excluding minimum school days (9 days of systematically missing data), and as such, data from 19 school days were included in the feasibility study. This data was then scrutinized for ‘adherence’, and only days on which both teacher and student paired observations were obtained at least 90% of the time were included. For example, when students were absent from school, or teachers were unexpectedly pulled for a meeting, this accounted for unsystematic loss of data. This resulted in 13 days (68% adherence), with a total of 141 teacher-student paired observation which were included in the pilot analysis.

iSelfControl was scheduled to prompt for a total of 11 centers across an entire day of school instruction. However, students’ numerous afternoon activities outside of the classroom (away from the iSelfControl iPad devices) made systematic data collection unfeasible after the lunch center. Subsequently, only the first seven iSelfControl centers across each day’s morning between the hours of 8:30 A.M. and 12:00 P.M. were included in the final analyses. Total points for each center were aggregated at the daily level (i.e., the day’s average points for each of the seven centers) separately for teacher- and student-reported scores. These mean points from daily centers were then aggregated across the 13 days of paired data entries.

Qualitatively, the prototype application described above was well received by both the classroom staff and students, and was consistently used in the classroom over a 6-week period. Quantitatively, the data gathered demonstrated that iSelfControl provided scheduled opportunities for self-reflections that are an important adjunct to conventional cognitive-behavioral interventions in the classroom. Specifically, when asked if they liked using the app in the classroom, the primary classroom teacher and a teaching assistant selected, “Yes.” When asked if they liked using the app, 70% of the children said “Yes”; those who indicated “No” reported difficulties with logging in and saving their data. When asked if they believed using the app helped them stay on track, 70% of the children (generally the same children who indicated that they liked the app) reported that it helped them, with one child writing in “A lot!”

As mentioned earlier, this pilot was conducted in a unique school in which all students participate in a universally delivered contingency management program, and all teachers regularly deliver observational feedback every 30 minutes in efforts to best serve children with executive function difficulties. Of note, this is a cumbersome and labor-intensive procedure, not easily reproduced in more typical educational settings. While the staff and students using this application found it acceptable and feasible to utilize, we caution that they are already participating in an intense behavior management system in which observational data is captured via paper and pencil. Future study should be conducted in more traditional educational settings and in longer intervals of time commensurate with typical instructional periods (e.g. 60–90 minutes).

### Improved Student Self-Awareness and Self-Regulation

**Fig 2:**
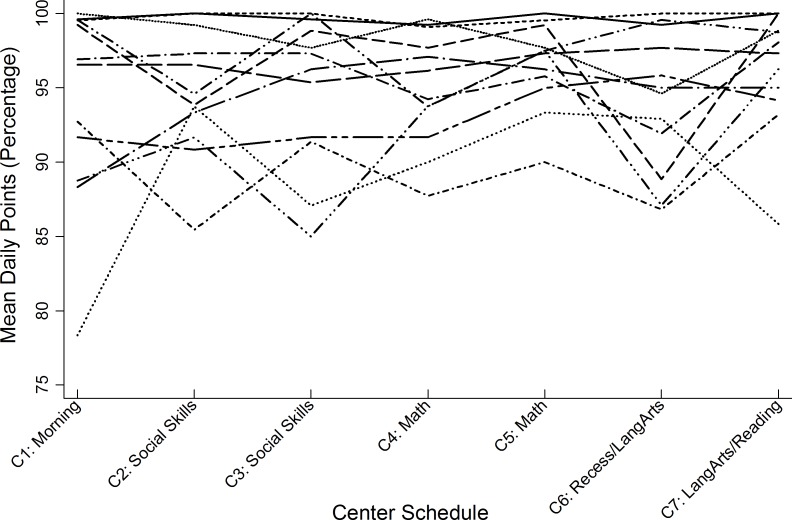
Teacher-reported mean daily center score percentages aggregated across 13 days.

**Fig 3:**
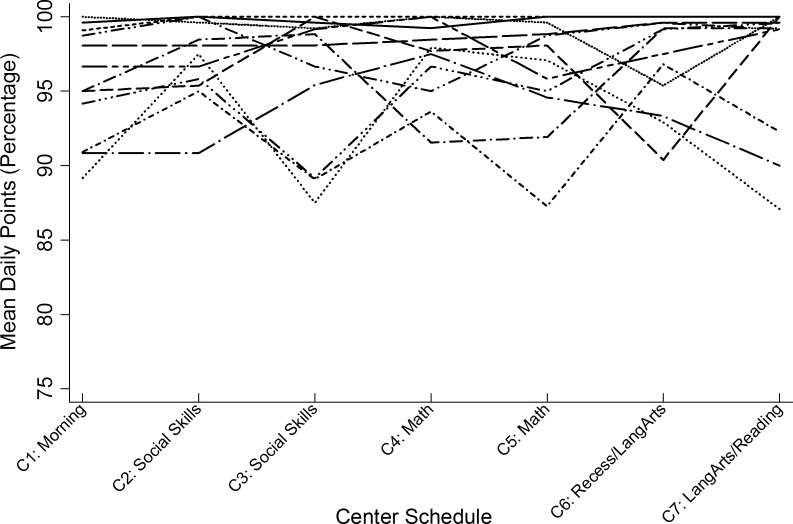
Student-reported mean daily center score percentages aggregated across 13 days.

Across the 12 dyads, the number of days in which points were entered for all seven of the same centers—30 minute periods of time—by both teacher and students ranged from 6 to 13 with a mean of 11.75 (*SD* = 1.88), resulting in a total of 84 paired center points. Figs 2 and 3 illustrate the percentage of teacher and student points (out of a possible 20 points), respectively. Results from the initial unconditional growth model indicated a significant non-zero mean center score of 19.06 (*SE* = .19, *p* < .001) for the overall sample, and that the non-significant positive slope did not show a linear trend or detectable change in the sample’s aggregated center scores over time (*β* = .04, *SE* = .02, *p* = .08). However, significant variance in the intercept (*β* = .37, *SE* = .17, *z* = 2.19, *p* < .05) indicated that mean center scores varied across dyads at baseline. The estimated intraclass correlation coefficient (ICC) indicated that 51% of the total variance in center scores was attributed to differences between dyads. Because the ICC also reflects the magnitude of dependency (autocorrelation) between repeated observations, the significant correlation between any pair of observations (e.g., centers 1 and 2, or 2 and 3) confirmed the need for multilevel modeling to explore between-dyad variance in mean center scores.

To evaluate the congruency between student and teacher scores, separate intercepts and slopes were estimated as fixed effects for both the student and teacher partners in the 12 dyads, while the only the intercepts of each dyad member were estimated as random effects. Table 1 presents the separate fixed-effects estimates and random-effects variance components of this model. Results indicated a significant non-zero mean score of 19.24 for students (*p* < .001) and a significant non-zero mean score of 18.88 for teachers (*p* < .001) at initial baseline (center 1). There was no significant effect of time on either student (*p* = .28) or teacher (*p* = .13) reports of mean daily center scores. Student and teacher trajectories were directly tested for differences in initial mean scores and rate of change in a re-parameterized model that constrained intercepts and slopes to be the same across dyad members. Results revealed that, at the first iSelfControl center of any given day, students reported significantly higher mean scores compared to teacher-reported scores (*F* (153) = 4.92, *p* < .05). In contrast, the degree of change in students’ daily scores across the repeated centers was not significantly different from the rate of change in corresponding teacher scores (*F* (153) = .16, *p* = .70). In sum, the trajectories of student and teacher scores remained relatively stable across the seven time points over the course of 13 days and did not statistically differ from each other, as evidenced by similar slope estimates (see Table 1). These differences were not accounted for by the degree of students’ familiarity with the classroom contingency point system, reflected as the length of time students were enrolled in the school program.

**Table 1:** Estimates of Fixed and Random Effects for Student and Teacher Center Scores.

					*CI*_95_	
*Fixed effects*	Estimate	*SE*	*t*	*p*	Lower	Upper
Intercept (level at center 1)						
Student	19.24	.18	106.04	< .001	18.86	19.62
Teacher	18.88	.24	79.81	< .001	18.38	19.39
Slope						
Student	.03	.03	1.09	.28	-.03	.09
Teacher	.05	.03	1.54	.13	-.01	.11
					*CI*_95_	
*Random effects variance components*	Estimate	*SE*	*z*	*p*	Lower	Upper
Level 2 (between-dyads)						
Student intercept	.26	.13	2.02	.02	.10	.67
Teacher intercept	.51	.24	2.14	.02	.20	1.28
Student-teacher intercept covariance	.33	.16	2.04	.02	.01	.65
Level 1 (within-dyads)						
Student residual	.29	.05	5.96	< .001	.21	.40
Teacher residual	.35	.06	5.96	< .001	.25	.48
Student-teacher residual covariance	.44	.10	4.55	< .001	.23	.61

As shown in Table 1, the random effects estimates for the separate intercepts indicated that there was significant variation in student scores (*p* < .05) and in teacher scores (*p* < .05) across dyads at initial baseline. The estimated values and confidence intervals suggested that there was more variation across teacher scores than student scores at the start of the day, and this variability in teacher scores was greater across dyads than within dyads. The covariance between intercepts was significant (*p* < .05) with a positive correlation of .91, suggesting that there was a high degree of similarity between student and teacher scores at the start of the day. Significant within-dyad variation in scores (residual correlations) that was not accounted for by time (*p* < .001) indicated that if the teacher in a particular dyad reported a high baseline score with a particular student, that student tended to concurrently report a high baseline score.

**Fig 4:**
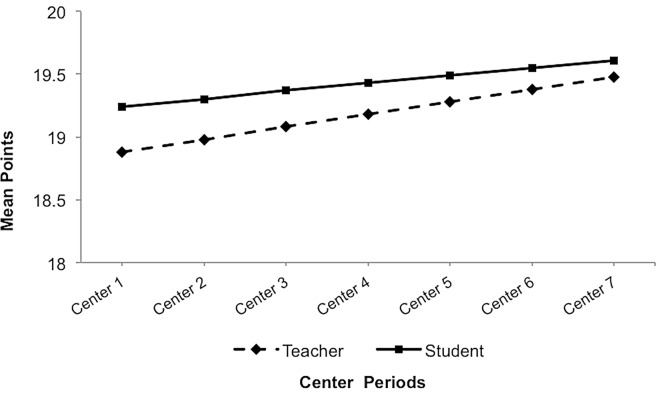
Predicted teacher and student daily mean center scores plotted at each time point across 13 days. Trajectories for each dyad for illustration (not referenced in text).

Although the average rate of change (or lack thereof) in student and teacher scores were similar over time, mean-level differences in scores at the start of any given day suggested that the congruency between predicted student and teacher scores at each subsequent center should be explored. Time was re-centered such that the intercept was shifted to a specified time point (i.e., iSelfControl center) to represent the initial baseline mean score when time = 0. Fig 4 displays the aggregated teacher and student mean scores at each center. Results from a series of separate mixed models revealed that there were significant differences between the mean center scores from student and teacher entries at each iSelfControl center (all *p’*s < .05) except at center 7 (*p* = .06), which was the final time point of each day. In sum, on any given day across the 13-day period, students tended to evaluate their classroom behaviors more positively and entered higher center scores for themselves compared to corresponding teacher scores. This difference between students and their teacher across dyads was detected at the first center of the day and continued across the next five consecutive centers until the last center of the day, in which mean scores were no longer statistically different. Given the nature of the program and that it is designed for children with disorders of executive function, it is not surprising that the students demonstrated relatively weak self-awareness in the beginning of the trial period. It is notable, however, that self-awareness gradually improved across the span of each day and over the period of the trial.

### Informing Classroom Behavior Management of *ADHD*

Individual differences in the objectivity of student’s perceptions of their points earned, as measured by degree of departure from points recorded by the classroom teacher, may be used to suggest individualized intervention strategies. For example, some students showed a tendency to consistently score themselves very highly irrespective of teacher recorded scores, while others seemed to consistently underestimate their capacities for self-regulation. The former group might need to reflect on their self-regulatory progress more often, while the latter might need more frequent prompts to consider their achievements.

Of note, the universal nature of the intervention program in place and the brevity of the pilot study, likely diminished the sensitivity of the applications ability to detect significant change in behavioral improvement, in terms of differences in observed scores over time. Specifically, the overall teacher and student scores were close to ceiling likely due to the significant behavioral supports in place in the specialized setting. While the application did capture some of the teacher/student discrepancies, of interest, it successfully captured significant variations across the centers. Additionally, due to resource limitations, the pilot version of iSelfControl was not programmed to provide pre-emptive warnings, individualized messages, or questions to guide self-reflection, strategies thought to contribute to improved self-regulation of behavior over time.

Moreover, we found that the application identifies differences in self-perception: some students appeared to have had little insight into the quality of their behavior (consistently estimating that they had earned the maximum number of points, irrespective of verbal feedback from the teacher to the contrary) while other students estimated their performance more harshly than their teacher observed/recorded. This information may suggest that the application is more beneficial for some students than for others, and that response may be moderated by identifiable co-variates. For both types of responses, students might benefit from gaining a more realistic self-perception, and iSelfControl could be used to encourage students to compare their estimations to the observations recorded by their teacher, which could lead to increased opportunities throughout the day to reflect on reasons for these discrepancies through the use of customized messages.

## Conclusion

The success of this preliminary investigation prompts us to consider additional features for iSelfControl, such as pre-emptive warnings prior to students entering instructional periods in which they have exhibited particular difficulties.

The next steps in this research will include further development of iSelfControl as well as the evaluation of a long-term intervention in both special education and general education settings and with control conditions (i.e. wait-list groups and traditional paper/pencil procedures). Furthermore, while this pilot study lacks sufficient statistical power to draw conclusions about diagnostic and demographic factors as potential covariates, future studies should include larger numbers of children both with and without behavioral health disorders. Similarly, this pilot study was conducted on archival data collected in the course of clinical programming implemented at a unique laboratory school setting in which children were already accustomed to a universal token economy system. It remains to be seen if this additional strategy in fact enhanced the children’s response to the program in place. Additionally, the generalizability of this tool to more typical settings needs to be examined. Future studies should address the need for experimental controls and generalizability across settings and populations. Despite these limitations, the preliminary findings suggest that iSelfControl could support the improvement of self-awareness and self-regulation for children with challenges related to disorders of executive function.
